# Baseline assessment of waste management practices and opportunities for smart waste technologies in traditional markets in Indonesia

**DOI:** 10.3389/fpubh.2026.1829762

**Published:** 2026-05-28

**Authors:** Aria Gusti, Defriman Djafri

**Affiliations:** Faculty of Public Health, Universitas Andalas, Padang, Indonesia

**Keywords:** environmental health, market sanitation, municipal solid waste, smart waste technologies, traditional markets, waste management practices

## Abstract

Inadequate waste management in traditional markets remains a persistent challenge in many low- and middle-income settings, with important implications for environmental sanitation and public health. This study assessed vendor-level waste management practices and identified context-specific opportunities for integrating smart waste technologies. A cross-sectional analytical study was conducted between June and August 2022 in three traditional markets in Indonesia representing urban, secondary city, and coastal contexts. Data were collected from 288 vendors using structured interviews and analyzed using descriptive and inferential statistics, including chi-square tests. Significant differences were observed across markets in access to waste disposal facilities, waste storage practices, disposal methods, and wastewater management systems (*p* < 0.05). While access to disposal sites was relatively high in some locations, waste segregation facilities were largely absent. Uncovered containers and informal storage methods were widely used, and variations in disposal and drainage systems reflected differences in infrastructure and service provision. These findings highlight system-level gaps in waste management practices that may contribute to environmental health risk pathways. Smart waste technologies, such as IoT-enabled bins and digital monitoring systems, offer opportunities to improve waste collection efficiency and sanitation; however, their implementation must align with local infrastructure and governance conditions. This study provides baseline empirical evidence to inform context-sensitive strategies for improving waste management in traditional markets.

## 1 Introduction

Traditional markets are critical components of urban food systems in many low- and middle-income countries, yet they often lack adequate waste management infrastructure. High volumes of organic and inorganic waste generated from daily trading activities, combined with limited waste storage, segregation, and collection systems, create conditions that may compromise environmental sanitation. These challenges are particularly relevant in rapidly urbanizing settings, where waste management systems struggle to keep pace with increasing waste generation (1). As a result, traditional markets are increasingly recognized as environments where inadequate waste management may contribute to environmental health risks.

Poorly managed waste in urban environments has been associated with environmental contamination and increased exposure to microbial and chemical hazards (2, 3). In addition, waste accumulation may create conditions that facilitate vector proliferation and disease transmission, particularly in densely populated settings (4, 5). Open burning of waste further contributes to air pollution and adverse health outcomes by releasing toxic emissions (6, 7). These risks are amplified in traditional market environments, where continuous food waste generation and high levels of human interaction increase the potential for environmental contamination (8–11).

Despite these challenges, waste management systems in many traditional markets remain constrained by limited infrastructure, inadequate waste segregation practices, and irregular waste collection services. In such settings, waste management is often shaped by complex interactions between municipal service provision, informal management systems, and vendor-level behaviors (12). These conditions highlight the need for innovative approaches to improve waste management practices in traditional markets.

In recent years, smart waste management technologies—including Internet of Things (IoT)-enabled bins, sensor-based monitoring systems, and data-driven collection optimization—have been increasingly explored as tools to improve waste management efficiency and sustainability. IoT-based systems enable real-time monitoring of waste levels and support more efficient collection scheduling (13, 14), while advances in artificial intelligence and data analytics have improved route optimization, waste sorting, and decision-making in municipal waste systems (15, 16). These approaches are increasingly integrated into broader smart city initiatives.

However, the implementation of such technologies in traditional market environments remains limited, partly due to insufficient baseline data on existing vendor-level waste management practices. Many studies focus on technological development or city-scale systems, while empirical evidence on how waste is managed in traditional market settings remains scarce.

However, existing studies on waste management in traditional markets remain limited, particularly in Southeast Asian contexts, and rarely examine vendor-level practices within informal system dynamics. In addition, comparative evidence across different geographical contexts of traditional markets remains limited. This gap constrains the development of context-sensitive interventions that align with local infrastructure, governance conditions, and operational realities.

Therefore, this study aims to assess waste management practices among vendors in traditional markets across three locations in Indonesia, representing different geographical contexts: Padang City, Payakumbuh City, and Air Bangis. The study examines access to waste disposal facilities, waste storage conditions, waste disposal methods, and sanitation systems to provide a baseline understanding of current waste management practices.

To guide the analysis, this study adopts a systems-based conceptual framework that conceptualizes waste management in traditional markets as a socio-technical system shaped by interactions among governance structures, infrastructure availability, market context, and vendor-level practices (17, 18). Within this framework, waste management practices are treated as observable system conditions rather than direct measures of environmental or health outcomes. Environmental health risks are therefore interpreted as potential pathways informed by existing literature, rather than as directly measured effects (6).

This systems perspective also incorporates feedback mechanisms, whereby environmental conditions and system performance may influence governance responses, service provision, and future waste management practices. Smart waste technologies are positioned as potential intervention components within this system, whose effectiveness depends on alignment with local infrastructure, governance capacity, and operational conditions (15). This approach enables a more context-sensitive understanding of how waste management practices can be improved in traditional market environments.

**Figure 1:**
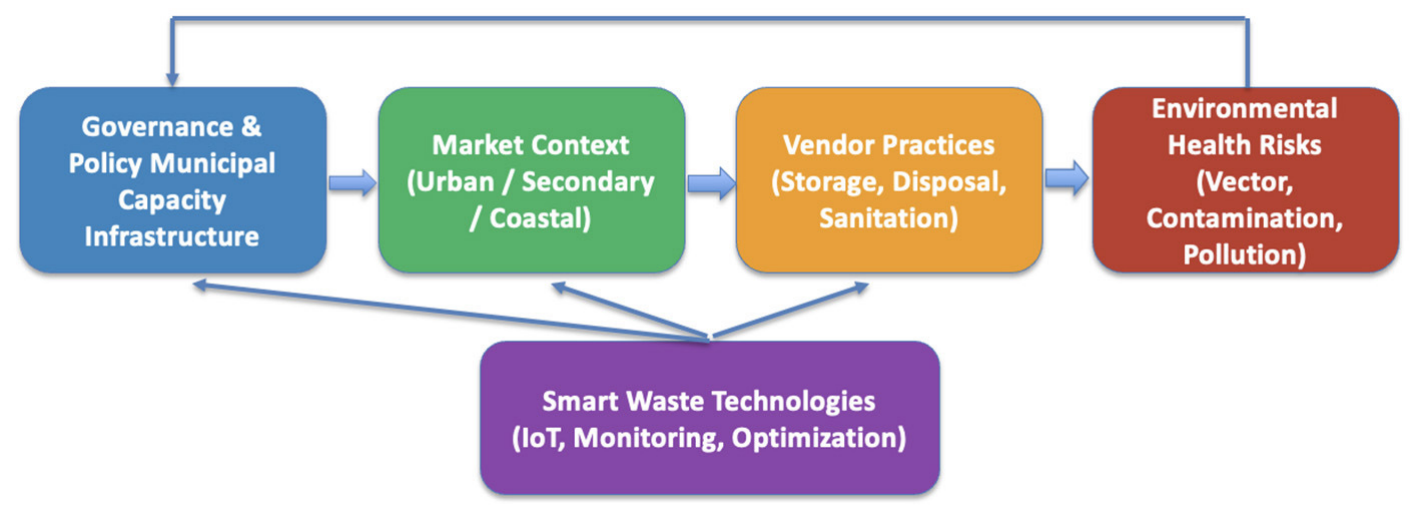
Systems-based conceptual framework illustrating the interactions between governance, infrastructure, market context, vendor waste management practices, and environmental health risks, with smart waste technologies positioned as an intervention layer within the system.

The conceptual framework presented in Figure 1 illustrates these interconnected relationships and emphasizes the importance of adopting a systems-oriented approach to understanding and improving waste management in traditional market environments.

This study is designed as a baseline comparative assessment to characterize existing waste management conditions across different market contexts, rather than to test causal relationships, and to contribute novel empirical evidence on vendor-level practices in Southeast Asia, a context that remains underexplored in the literature.

## 2 Methods

### 2.1 Study design and setting

This study employed a cross-sectional analytical design to assess waste management practices in traditional markets and to examine differences across market contexts. While the cross-sectional approach provides a snapshot of existing conditions, the study incorporates both descriptive and inferential analyses to enable statistical comparison between study sites. Data were collected between June and August 2022. This study did not include direct environmental measurements; instead, it focused on assessing waste management practices and system conditions.

The study was conducted in three traditional markets located in different geographical contexts in Indonesia: Nanggalo Market in Padang City, Ibuh Market in Payakumbuh City, and Air Bangis Market in West Pasaman. These markets were purposively selected to represent variations in urban characteristics, market activities, and access to municipal waste management services.

Nanggalo Market is located in Padang City, a major urban center in West Sumatra Province characterized by relatively higher population density, greater commercial activity, and more developed municipal waste management infrastructure. Ibuh Market is situated in the secondary city of Payakumbuh, where waste management systems are developing but may not yet fully meet the growing demand. Air Bangis Market is a coastal market in West Pasaman Regency, where waste management infrastructure is more limited and operational conditions differ from those in urban settings.

By comparing waste management practices across urban, secondary city, and coastal markets, this study aims to identify statistically significant differences in infrastructure, practices, and sanitation systems, and to explore associations among key variables using inferential statistical methods. This approach allows the study to move beyond descriptive comparisons and provide analytically grounded insights into variations in waste management practices across different market contexts. The comparative descriptive approach is appropriate for identifying baseline variations and system-level patterns across different market contexts.

### 2.2 Study population and sampling

The study population consisted of market vendors operating in three selected traditional markets: Nanggalo Market (Padang City), Ibuh Market (Payakumbuh City), and Air Bangis Market (West Pasaman). Vendors were selected as the primary unit of analysis because their daily trading activities generate substantial waste and directly influence waste storage, handling, and disposal practices within the market.

A non-probability sampling approach (convenience sampling) was employed. Vendors who were actively conducting trading activities and present at the time of data collection were invited to participate. Eligibility criteria included vendors who routinely generate waste as part of their activities, such as food vendors, vegetable sellers, fish sellers, and other traders handling perishable goods.

A total of 288 vendors were included in the study, with 96 vendors recruited from each market. The equal allocation across markets was intended to ensure balanced representation for comparative analysis rather than to reflect proportional sampling of the underlying vendor populations. Due to the absence of an official or up-to-date vendor registry in each market, a formal sampling frame could not be established.

Respondents were approached directly at their stalls, and those who consented were included in the study. This approach allowed for the collection of practical, real-time information on waste management practices; however, it may introduce selection bias, as vendors absent during the survey period or unwilling to participate were not captured.

A formal sample size calculation or power analysis was not conducted prior to data collection. Instead, the sample size was determined pragmatically based on field feasibility and the aim of achieving comparable sample sizes across markets to support statistical comparisons.

Given these considerations, the findings should be interpreted as reflecting observed practices among participating vendors rather than as statistically representative of the entire vendor population in each market and therefore may not fully represent all vendors within each market. Future studies are recommended to employ probability-based sampling methods and develop comprehensive vendor registries to improve representativeness and enable more robust generalization.

### 2.3 Data collection

Data were collected using a structured questionnaire developed to assess key components of waste management practices among market vendors. The instrument was developed based on a review of relevant literature on environmental sanitation and waste management in traditional market settings, as well as field observations conducted prior to data collection. The questionnaire included items on waste storage practices, waste disposal methods, access to waste management facilities, and sanitation conditions within the market environment. Most items were categorical (yes/no or multiple-choice options), allowing standardized responses across study sites.

To ensure content validity, the questionnaire was reviewed by experts in environmental health and waste management, who evaluated its relevance, clarity, and comprehensiveness. Minor revisions were made to improve the clarity and relevance of the items. Prior to the main data collection, the instrument was pilot-tested among 30 vendors in a comparable market setting to assess question clarity, flow, and feasibility of administration. Feedback from the pilot test was used to refine wording and improve response consistency.

The questionnaire's reliability was assessed using internal consistency measures, which indicated acceptable reliability for the study variables. Data collection was standardized through enumerator training and supervision to ensure data quality and completeness. Data were collected through face-to-face interviews conducted at vendors' stalls. Interviews were administered by trained field enumerators using a standardized questionnaire to ensure consistency across locations.

The questionnaire was administered in the local language (Bahasa Indonesia) to ensure participants' comprehension. The instrument was designed to capture both self-reported practices and observable conditions related to waste management and sanitation in the market environment.

### 2.4 Study variables

The study examined several variables related to waste management practices in traditional markets and environmental sanitation conditions. The primary contextual variable was the market location, categorized into three geographical contexts: an urban market (Padang), a secondary city market (Payakumbuh), and a coastal market (Air Bangis). This variable represents differences in environmental and infrastructural contexts that may influence waste management practices in traditional markets.

Waste management practice variables included vendor access to waste disposal facilities (V1), waste storage conditions (V2), waste disposal methods (V3), and wastewater management systems (V4) within the market environment. Access to waste disposal facilities refers to the availability of formal waste collection points or services within the market area. Waste storage conditions describe the types of containers vendors use to store waste generated during trading activities, including whether they are covered or uncovered. Waste disposal methods describe how vendors dispose of waste, such as at designated collection points, through open dumping, burning, or other informal practices. Sanitation or wastewater management systems describe how wastewater generated from market activities is managed, including drainage systems or other disposal mechanisms.

These variables were further interpreted from an environmental health perspective. For example, uncovered waste containers may facilitate vector breeding, improper waste disposal may increase environmental contamination, and inadequate drainage systems may contribute to water pollution and sanitation problems within the market environment.

### 2.5 Data analysis

Data analysis used both descriptive and inferential statistical methods to examine waste management practices across the three study locations. Descriptive statistics were used to summarize the distribution of waste storage conditions, waste disposal methods, and sanitation or drainage systems. Proportional distributions were calculated to describe the prevalence of observed practices.

Frequencies and proportions were calculated for each variable and stratified by market location (Padang, Payakumbuh, and Air Bangis) to enable direct comparison across study sites. These descriptive measures were used to characterize patterns of waste management practices and to identify variations across different market contexts.

To address differences across study sites, inferential statistical analysis was incorporated. Chi-square tests were performed to examine associations between market location and key categorical variables, including access to waste disposal facilities, waste storage practices, disposal methods, and wastewater management systems. These tests were used to determine whether observed differences across markets were statistically significant. Effect sizes were calculated to assess the strength of associations identified in chi-square tests.

Bivariate analyses were further conducted to explore associations between selected variables, particularly the relationship between access to waste disposal facilities and waste disposal practices. This approach allowed the identification of factors associated with variations in waste management practices while maintaining robustness, given the dataset's structure. Accordingly, the results indicate associations between variables and should not be interpreted as evidence of causal relationships.

Multivariate analysis, such as logistic regression, was considered to examine determinants of waste management practices. However, it was not performed due to limited variability in several key variables, including the high prevalence of certain outcomes and low variation in some predictors, which may lead to unstable or unreliable model estimates. Therefore, a parsimonious analytical approach was adopted to ensure valid and interpretable results.

Confidence intervals and advanced clustering analyses were not included, as the primary objective of the study was to examine comparative differences and associations across market contexts rather than to develop predictive or classification models. These approaches are recommended for future studies with more heterogeneous data and larger sample sizes.

In addition, a conceptual mapping approach was employed to identify system-level gaps and opportunities for integrating smart waste management technologies. Observed patterns in infrastructure availability and waste management practices were used to inform potential intervention areas, linking empirical findings with broader system-level considerations.

### 2.6 Ethical considerations

Participation in this study was voluntary, and respondents were informed of the research's purpose and objectives prior to data collection. Verbal informed consent was obtained from all participants before the interviews were conducted.

The questionnaire collected information related to waste management practices in traditional markets and did not involve sensitive personal data. All collected data were anonymized and analyzed in aggregate to ensure the confidentiality and privacy of respondents.

The study adhered to general ethical principles for research involving human participants, including voluntary participation, informed consent, and protection of respondent confidentiality.

## 3 Result

### 3.1 Overview of waste management practices across study locations

This study assessed waste management practices among vendors in three traditional markets in Indonesia. These markets represent different geographic contexts, including an urban market, a secondary city market, and a coastal market.

The results describe variations in vendors' access to waste disposal facilities, waste storage conditions, waste disposal practices, and sanitation or drainage systems within the market environments.

Overall, statistically significant differences were observed across the three markets in multiple domains of waste management. Chi-square tests indicated significant variations in access to waste disposal facilities, waste storage practices, disposal methods, and wastewater management systems (all *p* < 0.05).

These findings indicate that waste management practices differ systematically across market contexts, with variations observed in both infrastructure availability and vendor practices, as summarized in Table 1. This table highlights distinct patterns across urban, secondary city, and coastal market settings.

**Table 1:** Summary of waste management conditions across study markets.

Domain	Padang (urban)	Payakumbuh (secondary city)	Air Bangis (coastal)	Key comparative pattern
Waste disposal facility availability	Moderate access to disposal sites; limited collection services	Moderate access; slightly higher reliance on informal disposal	High access to disposal sites; minimal formal collection service	Facility access highest in coastal market, but service support uneven
Waste storage practices	Pre-dominantly uncovered containers	Pre-dominantly uncovered containers	More varied (plastic bags, sacks)	Urban and secondary markets show higher reliance on uncovered storage
Waste disposal methods	Primarily disposal at designated sites	Mixed methods, including informal disposal	Pre-dominantly collection by waste collectors	Distinct disposal patterns across market contexts
Wastewater management systems	Mixed (closed and open drainage)	Pre-dominantly open drainage	Pre-dominantly closed drainage	Infrastructure quality varies by market context

### 3.2 Waste disposal facilities

As shown in Table 2, waste disposal facilities varied across the three study locations. A chi-square test showed a statistically significant difference in facility access among markets (χ^2^ = 44.87, *p* < 0.001), with a moderate effect size (Cramér's *V* = 0.279), indicating a meaningful association between market context and facility availability.

**Table 2:** Availability of waste disposal facilities in traditional markets.

Waste disposal facility	Padang	Payakumbuh	Air Bangis	*P*
The waste disposal site easily accessible	60 (62.5%)	60 (62.5%)	93 (96.9%)	< 0.001
Separate bins for dry and wet waste available	2 (2.1%)	0 (0.0%)	2 (2.1%)	
Waste transportation services are available	34 (35.4%)	36 (37.5%)	1 (1.0%)	

Vendors in Air Bangis reported more frequent easy access to disposal sites than those in Padang and Payakumbuh, suggesting relatively better baseline facility availability in the coastal market.

In contrast, waste segregation facilities were largely absent across all markets, indicating a systemic gap in waste management infrastructure rather than location-specific differences.

Patterns of waste collection services also differed across markets, with Padang and Payakumbuh showing greater reliance on collection services, whereas they were almost absent in Air Bangis. These results indicate substantial variation in service availability across markets, despite relatively high reported accessibility of disposal sites in some locations.

### 3.3 Waste storage practices

As shown in Table 3, waste storage practices differed significantly across the three study locations (χ^2^ = 57.26, *p* < 0.001), with a moderate effect size (Cramér's *V* = 0.315), indicating a meaningful association between market context and storage practices.

**Table 3:** Waste storage practices among vendors in traditional markets.

Waste storage Practices	Padang	Payakumbuh	Air Bangis	*P*
Uncovered container	60 (62.5%)	52 (54.2%)	22 (22.9%)	< 0.001
Covered container	6 (6.3%)	0 (0.0%)	0.0%	
Plastic bag	14 (14.6%)	26 (27.1%)	29 (30.2%)	
Basket	10 (10.4%)	9 (9.4%)	19 (19.8%)	
Sack	6 (6.3%)	9 (9.4%)	26 (27.1%)	

Uncovered containers were more commonly used in Padang and Payakumbuh, whereas vendors in Air Bangis reported a more varied range of storage methods, including plastic bags and sacks. This pattern suggests that storage practices in urban and secondary markets are more standardized but may rely on less protective methods. Across all markets, the use of covered containers remained minimal, indicating a persistent gap in safe, hygienic waste storage practices.

These findings indicate that waste storage practices differ significantly across markets, with uncovered containers dominating in urban and secondary settings, while more varied storage methods are observed in the coastal context.

### 3.4 Waste disposal methods

As shown in Table 4, waste disposal methods varied significantly across the three study locations (χ^2^ = 66.64, *p* < 0.001), with a moderate-to-strong effect size (Cramér's *V* = 0.340), indicating substantial differences in disposal practices across market contexts.

**Table 4:** Waste disposal methods in traditional markets.

Waste disposal methods	Padang	Payakumbuh	Air Bangis	*P*
Dumped in nearby bushes	10 (10.4%)	17 (17.7%)	2 (2.1%)	< 0.001
Disposed at designated disposal sites	52 (54.2%)	26 (27.1%)	13 (13.5%)	
Disposed into drainage channels	0 (0.0%)	9 (9.4%)	4 (4.2%)	
Collected by waste collectors	34 (35.4%)	44 (45.8%)	77 (80.2%)	

Vendors in Air Bangis pre-dominantly relied on waste collection services, whereas vendors in Padang more frequently disposed of waste at designated disposal sites. In contrast, Payakumbuh exhibited a more heterogeneous pattern, including both formal and informal disposal practices.

Informal disposal methods, such as dumping waste in nearby bushes, were more commonly observed in Padang and Payakumbuh, while disposal into drainage systems was reported in Payakumbuh and, to a lesser extent, in Air Bangis, but not in Padang. These findings indicate distinct, context-specific baseline disposal patterns across market settings.

### 3.5 Wastewater disposal systems

As shown in Table 5, wastewater disposal systems differed significantly across the three study locations (χ^2^ = 34.90, *p* < 0.001), with a moderate effect size (Cramér's *V* = 0.246), indicating a meaningful association between market context and wastewater management systems.

**Table 5:** Wastewater disposal systems in traditional markets.

Wastewater disposal systems	Padang	Payakumbuh	Air Bangis	*P*
Piped wastewater system	2 (2.1%)	0 (0.0%)	0 (0.0%)	< 0.001
Closed drainage system	54 (56.3%)	35 (36.5%)	73 (76.0%)	
Open drainage system	40 (41.7%)	61 (63.5%)	23 (24.0%)	

Closed drainage systems were more commonly reported in Air Bangis and Padang, whereas Payakumbuh relied more on open drainage systems, reflecting differences in sanitation infrastructure across markets.

Open drainage was also present in Padang and, to a lesser extent, in Air Bangis, indicating variability in sanitation conditions even within markets with limited infrastructure. In contrast, piped wastewater systems were rarely reported across all locations, suggesting limited adoption of more advanced wastewater management systems.

Overall, these findings demonstrate substantial differences in baseline wastewater management systems across the three markets, reflecting variations in sanitation infrastructure and operational conditions.

## 4 Discussion

### 4.1 Waste management challenges in traditional market environments

Building on these findings, this study highlights how variations in infrastructure availability and operational conditions shape waste management practices across traditional market contexts. The environmental health risks discussed in this study should be interpreted as potential pathways based on observed conditions, rather than directly measured outcomes. These findings should be interpreted as indicating associations rather than causal relationships, as the study design does not allow causal inference.

The findings reveal several important gaps in waste management practices within traditional market environments. Although waste disposal facilities were available in some markets, waste segregation systems were largely absent, and many vendors relied on uncovered containers or informal storage materials such as plastic bags and sacks. In addition, sanitation infrastructure, including piped wastewater systems and closed drainage systems, was limited in most market areas.

Similar challenges have been reported in studies examining municipal solid waste management systems in low- and middle-income countries. Research has shown that waste management systems in developing regions often face infrastructure deficits, limited waste segregation, and weak institutional oversight, leading to inefficient waste collection and environmental contamination (17–21). These structural constraints are particularly evident in informal commercial environments such as traditional markets, where waste generation is continuous, and waste management services may be insufficient.

Traditional markets are increasingly recognized as environmental health hotspots due to interactions among high population density, food-handling activities, and large volumes of organic waste generated during daily trading. Organic waste from vegetables, fruits, meat, and fish products can rapidly decompose in warm climates, attracting flies, rodents, and other disease vectors when not properly managed. Studies conducted in urban markets in Africa and Asia have demonstrated that inadequate waste management and sanitation practices can contribute to microbial contamination of food products and increase the risk of foodborne diseases (8, 11, 22). These findings are consistent with the present study's findings, which observed widespread use of uncovered waste containers and informal waste storage practices among market vendors.

The high prevalence of uncovered waste containers observed in this study indicates suboptimal waste storage practices. Such conditions may facilitate exposure of organic waste to the surrounding environment. Previous studies examining sanitation practices in food markets have shown that poorly managed organic waste may create favorable conditions for flies and rodents, which can serve as vectors for disease transmission (10, 23). In addition, the decomposition of organic waste may generate leachate, odors, and greenhouse gas emissions, contributing to environmental pollution and climate change (20, 24, 25). However, this study did not directly measure vector presence or contamination levels; therefore, these risks should be interpreted as potential rather than observed outcomes.

The findings of this study are also consistent with research on Southeast Asian cities, where traditional markets remain an important component of urban food systems but often lack adequate sanitation infrastructure. Studies conducted in Indonesia, Thailand, and Vietnam have reported similar challenges, including limited waste segregation systems, inadequate drainage infrastructure, and insufficient waste collection services in traditional market environments (9, 18, 26). These conditions often lead to the accumulation of organic waste and increase environmental health risks in densely populated market areas.

The variation observed across the three markets in this study also highlights the influence of geographical context on waste management practices. For example, the higher reliance on waste collectors observed in Air Bangis suggests that informal or community-based waste collection systems may play an important role in coastal communities where formal municipal waste services are limited. Similar patterns have been observed in small cities and coastal regions in Southeast Asia, where informal waste collectors often complement municipal waste management systems (18, 26). These findings highlight the importance of considering local socio-economic contexts when designing waste management interventions in traditional market environments.

Recent research also emphasizes that improving waste management in traditional markets requires integrated approaches that combine infrastructure development, institutional support, and vendor behavioral change. Studies have shown that vendor awareness, availability of waste containers, and regular waste collection services are key factors influencing waste management practices in market environments (9, 27). These findings support the present study's conclusion that improving waste management systems in traditional markets requires coordinated interventions involving market management authorities, local governments, and vendors.

### 4.2 Opportunities for smart waste management technologies

The opportunities for smart waste management technologies identified in this study were derived from observed system-level gaps across the three study markets (Tables 1–5). Rather than proposing technological solutions *a priori*, this study adopts an evidence-informed approach by mapping empirical findings to potential technological options and their implementation prerequisites, including infrastructure availability, governance capacity, and resource requirements. This approach maintains the analysis's exploratory nature while grounding it in observed conditions.

For example, the limited availability of waste segregation facilities and the widespread use of uncovered or non-standardized storage methods (Table 3) indicate operational deficiencies at the vendor level. These conditions suggest the potential for IoT-enabled smart bins with segregation guidance features to improve waste-handling practices. However, effective implementation would require supporting conditions such as reliable electricity supply, user acceptance, and maintenance capacity.

Similarly, variations in access to disposal facilities and waste collection services (Tables 2, 4) suggest inefficiencies in collection systems. These gaps may be addressed through digital monitoring platforms and route optimization tools. Nevertheless, such technologies depend on adequate digital infrastructure, institutional coordination, and operational funding mechanisms.

Differences in wastewater management systems (Table 5), particularly the reliance on open drainage in some markets, further highlight infrastructural variability that may influence the applicability of technology-based interventions. These findings suggest that smart waste technologies should be adapted to local infrastructure conditions rather than applied uniformly.

Recent studies demonstrate that IoT-enabled waste monitoring systems and AI-based optimization models can improve collection efficiency and reduce operational costs in urban waste management systems (15). Similarly, digital waste tracking and smart bin technologies enhance real-time monitoring and decision-making in waste systems. (28–32) In addition, integrated smart waste management frameworks emphasize the importance of aligning technological solutions with governance capacity and infrastructure readiness to ensure effective implementation (31). Advances in artificial intelligence and data analytics further support optimized collection routing and improved waste segregation processes (33, 34) while digital platforms in food supply chains contribute to better waste tracking and reduction (35, 36).

However, it is important to emphasize that this study did not directly assess key feasibility dimensions related to smart waste technology implementation, including electricity availability, digital infrastructure, governance capacity, cost considerations, and stakeholder readiness. Therefore, the identification of technological opportunities in this study should be interpreted as conceptually informed by empirical system gaps, rather than as evidence of implementation feasibility.

In this context, the contribution of the present study is to provide a baseline, context-specific assessment of waste management conditions in traditional markets, which can serve as a foundation for future research assessing the technical, economic, and institutional feasibility of implementing smart waste technologies in similar settings.

### 4.3 Global relevance of waste management in traditional markets

Improving waste management in traditional markets is not only a local sanitation issue but also a global public health concern. Traditional and informal markets remain essential components of food systems in many low and middle-income countries, providing affordable access to food and supporting livelihoods for millions of small-scale vendors. However, the rapid growth of urban populations and expanding food distribution networks have significantly increased waste generation in these markets.

Global studies on municipal solid waste management have shown that commercial food markets contribute substantially to urban organic waste streams, particularly in cities across Asia, Africa, and Latin America. (20, 21, 26) When poorly managed, waste generated in market environments can contribute to environmental pollution, greenhouse gas emissions, and the spread of infectious diseases. In addition, waste accumulation in urban food markets may affect surrounding residential communities by contaminating water systems, attracting disease vectors, and degrading environmental quality.

From a public health perspective, strengthening waste management systems in traditional markets is therefore essential for improving food safety, environmental sanitation, and urban health outcomes. Integrating innovative waste management approaches, such as digital monitoring systems and smart waste technologies, may create new opportunities to improve waste management efficiency while supporting sustainable urban development. Consequently, improving waste management practices in traditional markets should be considered an important component of global efforts to achieve sustainable urban sanitation and environmental health goals.

### 4.4 Study limitations

This study has several limitations that should be considered when interpreting the findings. Although inferential statistical tests (chi-square) were conducted, the analysis remains primarily descriptive, and differences between markets should be interpreted with caution. First, the cross-sectional design provides a snapshot of waste management practices at a single point in time and does not capture temporal variations in waste generation and management practices; therefore, the findings reflect associations rather than causal relationships. Second, the study focused primarily on vendor-level practices and observable infrastructure conditions and did not comprehensively examine broader institutional and governance factors that influence waste management systems in traditional markets.

In addition, this study did not directly measure environmental or public health outcomes, such as vector populations, contamination levels, or pathogen presence. Therefore, interpretations of environmental health risks should be understood as indicative of potential risk pathways informed by existing literature, rather than as directly observed effects.

Furthermore, this study did not assess key feasibility dimensions related to smart waste technologies, including digital infrastructure availability, energy access, governance readiness, and cost considerations. As a result, the discussion of technological interventions is based on identifying system-level gaps rather than on an empirical evaluation of implementation feasibility. Future studies should incorporate these dimensions to evaluate the practical applicability and scalability of technology-based waste management solutions in traditional market settings.

Despite these limitations, the study provides important baseline evidence on waste management practices in traditional markets and highlights opportunities for integrating smart waste management technologies to improve environmental sanitation and public health outcomes.

In addition, although the questionnaire was developed based on relevant literature, reviewed by experts, and pilot-tested prior to data collection, the study did not conduct extensive psychometric validation, such as formal construct validation or comprehensive reliability testing across all variables. Therefore, measurement error or reporting bias cannot be fully excluded. Future studies are recommended to employ more rigorous instrument validation procedures, including the use of standardized scales and reliability assessments, to improve measurement accuracy.

## 5 Conclusion

This study provides a context-specific, comparative assessment of waste management practices across three traditional markets in Indonesia and demonstrates statistically significant differences in infrastructure availability, storage practices, disposal methods, and sanitation systems across market contexts. The findings highlight persistent system-level gaps, particularly in waste segregation, storage conditions, and wastewater management, which may create potential environmental health risk pathways in traditional market environments. Addressing these challenges requires integrated approaches that combine improvements in infrastructure, service delivery, and vendor practices. While smart waste technologies offer promising opportunities to enhance waste monitoring and collection efficiency, their implementation should be context-sensitive and aligned with local system conditions. Overall, this study provides baseline empirical evidence to inform future research and policy efforts to develop sustainable, adaptive waste management strategies in traditional market settings.

## Data Availability

The raw data supporting the conclusions of this article will be made available by the authors, without undue reservation.
