# Supplementary material for: Baseline assessment of waste management practices and opportunities for smart waste technologies in traditional markets in Indonesia
Source: Front Public Health. 2026 May 28;14:1829762. doi: 10.3389/fpubh.2026.1829762 (PMC13253738; doi:10.3389/fpubh.2026.1829762)
Supplement: Supplementary file 1 [file Data_Sheet_1.pdf]

Research Questionnaire

Research Title: Sanitation and Pro-Environmental Behavior at Traditional Market

Peace be upon you, and may Allah mercy and blessings

We are a research team from the Faculty of Public Health at Andalas University, conducting research at traditional markets in West Sumatra. Our research focuses on the availability of sanitation facilities and on vendors' behavior when using them.

Please answer each statement based on your own opinions and circumstances, not based on the influence of others. Your answers will be kept confidential and will not influence anything. Your responses will be used solely for research purposes and for no other purpose.

Thank you for your help and cooperation.

Peace be upon you

Dr. Aria Gusti , SKM, M.Kes
Chief Researcher

R-ID*				

* filled in by researcher

IR	RESPONDENT IDENTITY		Answer
IR-1	Market	1. Nanggalo<br>2. Ibuh<br>3. Air Bangis	
IR-2	Sex	1. Male<br>2. Female	

A. Access to environmental sanitation facilities

Choose one of the answers to the following questions by marking (**X**) the answer that you think is most correct.

1. Clean water sources used :
   a. tap water
   b. Boreholes
   c. dug well
   d. Refill water

2. Do you have access to toilets at this market:
   a. Yes
   b. No

3. Temporary waste disposal site:
   a. Easy to reach
   b. Wet and dry trash bins are available
   c. Garbage collection equipment available

4. Types of wastewater drainage channels:
   a. Piping
   b. Closed channel
   c. Open channel

5. Hand washing facilities:
   a. Easy to reach location
   b. Equipped with soap
   c. Running water available

B. Pro-Environmental Behavior

Choose one of the answers to the following questions by marking (**X**) the answer that you think is most correct.

1. Types of waste storage facilities:
   a. Container without a lid
   b. Container with lid
   c. Plastic bags
   d. Basket
   e. Bag

2. Waste disposal methods:
   a. Thrown into nearby bushes
   b. Dispose of in the disposal bin provided
   c. Discharged into drains/drainage
   d. Collected by rubbish collectors

3. Distance from the nearest clean water source (m):
   a. $\leq$50
   b. 51-100
   c. $\geq$100

4. Distance from toilet:
   a. $\leq$50
   b. 51-100
   c. $\geq$100

5. Factors that reduce interest in using the toilet:
   a. Poor sanitary conditions
   b. Long distance
   c. Others, please specify
   ………
   ………
   …………………

= **Thank you for your participation** =
